# Going Forward and Back: The Complex Evolutionary History of the GPx

**DOI:** 10.3390/biology10111165

**Published:** 2021-11-12

**Authors:** Thomaz Stumpf Trenz, Camila Luiza Delaix, Andreia Carina Turchetto-Zolet, Marcel Zamocky, Fernanda Lazzarotto, Márcia Margis-Pinheiro

**Affiliations:** 1 Programa de Pós-Graduação em Biologia Celular e Molecular, Centro de Biotecnologia, Universidade Federal do Rio Grande do Sul (UFRGS), Porto Alegre 91509-900, Brazil; thomaz.trenz@ufrgs.br; 2 Graduação em Biotecnologia, Departamento de Biologia Molecular e Biotecnologia, Universidade Federal do Rio Grande do Sul (UFRGS), Porto Alegre 91509-900, Brazil; camila.delaix@ufrgs.br; 3 Programa de Pós-Graduação em Genética e Biologia Molecular, Departamento de Genética, Instituto de Biociências, Universidade Federal do Rio Grande do Sul (UFRGS), Porto Alegre 91509-900, Brazil; carina.turchetto@ufrgs.br; 4 Laboratory of Phylogenomic Ecology, Institute of Molecular Biology, Slovak Academy of Sciences, Dúbravská cesta 21, 84551 Bratislava, Slovakia; marcel.zamocky@savba.sk; 5 Department of Chemistry, Institute of Biochemistry, University of Natural Resources and Life Sciences, Vienna, Muthgasse 18, 1190 Vienna, Austria

**Keywords:** GPx, peroxidase family, evolution, selenocysteine, ROS

## Abstract

**Simple Summary:**

Glutathione peroxidases (GPxs) are considered as one of the main antioxidant enzymes, which reduce peroxides into less toxic compounds. This family of enzymes is found in most eukaryotic organisms, but it is highly divergent regarding its structure, catalytic mechanism, and substrate usage. Furthermore, it is still unclear how these enzymes are dispersed in the animal kingdom. Through robust phylogenetic and sequence analyses, we show that all GPx genes originated from a common ancestor and evolved independently across different kingdoms. In Metazoa, GPx genes expanded into three main groups before the rise of bilaterian animals, and they were further expanded in vertebrates. These expansions allowed GPx enzymes to diversify, not only structurally, but also functionally. Our study contributes to the understanding of how this abundant class of antioxidant enzymes evolved. The evolution of GPxs appears to be a continuous process, leading to the diversification of their functions.

**Abstract:**

There is large diversity among glutathione peroxidase (GPx) enzymes regarding their function, structure, presence of the highly reactive selenocysteine (SeCys) residue, substrate usage, and reducing agent preference. Moreover, most vertebrate GPxs are very distinct from non-animal GPxs, and it is still unclear if they came from a common GPx ancestor. In this study, we aimed to unveil how GPx evolved throughout different phyla. Based on our phylogenetic trees and sequence analyses, we propose that all GPx encoding genes share a monomeric common ancestor and that the SeCys amino acid was incorporated early in the evolution of the metazoan kingdom. In addition, classical GPx and the cysteine-exclusive GPx07 have been present since non-bilaterian animals, but they seem to have been lost throughout evolution in different phyla. Therefore, the birth-and-death of GPx family members (like in other oxidoreductase families) seems to be an ongoing process, occurring independently across different kingdoms and phyla.

## 1. Introduction

The rising concentration of molecular oxygen (O_2_) initially in the seawater, and later in the atmosphere, has dramatically changed how life has evolved on Earth [1]. Besides the formation of the ozone layer and the profound effect in the chemical element composition on oceans, oxygen promptly reacted with metals to produce reactive oxygen species (ROS) [2,3]. Most of these species can readily oxidize other molecules, such as lipids, proteins, and nucleic acids, causing damage and eventually leading to cell death.

Enzymatic and non-enzymatic molecules emerged as a way to circumvent the harm that ROS would cause. Some of them serve as baits for the highly reactive hydroxyl radical, and others can reduce the hydrogen peroxide (H_2_O_2_) into less toxic compounds [4]. One of the main antioxidant enzyme specificities is represented by a large glutathione peroxidase family (mostly abbreviated as GPx) [5].

GPx was the first selenoprotein described [5]. This enzyme is able to reduce hydrogen peroxide into water, using glutathione (GSH) as a reducing agent [6]. Most vertebrate glutathione peroxidases contain selenocysteine (SeCys) in their active site, and at least half of the vertebrate GPxs are homotetrameric proteins. These tetrameric enzymes, also known as classical GPx (cGPx), contain a dimer/tetrameric domain and were thought to be exclusive to vertebrates [7]. cGPx were better characterized in mammals, in which they comprise five subfamilies: GPx01 and its paralog GPx02, GPx03 and paralogs GPx05 and GPx06, the last two being exclusive to mammals [8]. Members of GPx05 are the only cGPxs that strictly contain cysteine (Cys) in its catalytic site, although a few GPx06, especially from rodents, also present this amino acid instead of SeCys [9]. Genes putatively encoding classical GPx in invertebrates were described in Crustacean and Mollusca elsewhere [10,11,12,13,14,15], but it is still unclear whether they are present in other animal phyla, and if cGPx share ancestrality with other animal GPxs [7]. 

The most intensively studied glutathione peroxidase is a monomeric enzyme, which comprises the GPx04 subfamily. It is found in many animals, including invertebrates, and it can reduce a broader range of substrates, other than hydrogen peroxide, into the water and their corresponding alcohols [16]. It also belongs to the once called phospholipid hydroperoxide GPx (PHGPx), a subgroup of monomeric GPx that can reduce phospholipids. It comprises all GPx found in fungi, bacteria, and plants [17]. Despite the presence of SeCys in the vertebrate GPx04, PHGPxs are mainly SeCys-independent, containing instead a Cys residue at the same critical position within the active site. This difference raises the question of whether SeCys was incorporated into GPx from vertebrates, or whether it was lost in other organisms [17].

The most recent vertebrate GPx described are GPx07 and its paralog GPx08—both target the endoplasmic reticulum (ER) lumen [18]. They are exclusively Cys-GPx enzymes which also lack the dimerization domain found in classical GPx. In addition, they are preferably reduced by the protein disulfide isomerase (PDI) and are involved in oxidative protein folding [8,19]. Orthologs of GPx07/08 were found in a few invertebrates [20,21], but little is known about the distribution of these enzymes in the animal kingdom. 

Plant and fungal GPxs are part of the PHGPx, reducing lipid peroxides and H_2_O_2_ [22,23], and most of them are monomeric [24,25]. They use almost exclusively thioredoxin as a reducing agent and hence were first described as GPX-Like enzymes (GPXL) [26]. Furthermore, they do not rely on one but two cysteines to catalyze the substrate reduction: the peroxidatic cysteine (Cys_p_), which directly interacts with the peroxide (forming the sulfenic acid intermediate), and the resolving cysteine (Cys_r_), which forms an intramolecular disulfide bond with the Cys_p_, releasing a molecule of water [27]. This mechanism resembles the catalytic system of peroxiredoxins, reinforcing the argument that plant and fungal GPxs are not true glutathione peroxidases. Despite the differences, they are proposed to originate from the same ancestor that gave rise to the animal GPx4 [7]. The mutation of the Cys-to-SeCys in citrus GPx showed a significant increase in its activity towards peroxides, indicating the amino acid substitution does not impair its peroxidase activity [28]; however, the activity of the mutated citrus GPx was dramatically lower than the vertebrate’s SeCys-GPx04, suggesting that their function has differences which are beyond this amino acid substitution.

There is little information about bacterial GPxs compared to the other groups mentioned above. Even so, they present both peroxidatic and resolving cysteines, and can use several reducing agents (including thioredoxin and GSH), as well as reduce different types of peroxides [29,30].

In this study, we evaluate the phylogenetic relationship among GPx encoding genes from different kingdoms of life, aiming to unveil how and when these proteins became so diverse regarding their structure, reducing agent, and catalytic mechanisms, giving a particular focus on animal GPxs. Based on our phylogenetic trees and sequence analysis, we propose that all GPx encoding genes share a monomeric common ancestor and that the SeCys amino acid was incorporated early in the evolution of the metazoan kingdom. Classical GPx and the Cys-exclusive GPx07 are present since non-bilaterian animals, but they were lost throughout evolution in different phyla. In addition, a model is proposed as a way to explain how GPx proteins independently evolved. 

## 2. Materials and Methods

### 2.1. GPx Sequence Search

GPx sequences were chiefly retrieved from NCBI (https://www.ncbi.nlm.nih.gov, accessed on 20 March 2021) and RedoxiBase (https://peroxibase.toulouse.inra.fr/, accessed on 15 October 2020) [31] databases. Human HsGPx01, 03, 04, 07, and *Escherichia coli* EcoGPx protein sequences were used as queries to search for GPx in other species, using the translated Basic Local Alignment Search Tool (tBLAST and BLASTp, available at https://blast.ncbi.nlm.nih.gov/Blast.cgi, accessed on 20 March 2021). Sequences from animals whose genomes were not deposited on NCBI were retrieved from their specific databases (Table S1). Matching sequences were only considered glutathione peroxidases if they contained the three GPx signature motifs: “G[K/R/S]x[L/V/C/S][I/L]I[V/E/T]NVA[S/T/A][E/Q/L/Y][C/U]G[L/T]T”, “LAFPCNQF” and “WNF(S/T)KF”, with some differences in non-animals GPx sequences [32,33]. Full-length protein sequences were used in all phylogenetic analyses, and partial sequences were only used to confirm the existence of a specific class of GPx in phyla whose genome sequences are scarce (Table S1). Classical GPx (cGPx) was defined by typical features indicating the presence of the dimer/tetramer domains [18,25].

Global GPx tree was constructed using representative species of major phyla from Bacteria (*Escherichia coli*, Gammaproteobacteria; *Mycoplasma fermentans*, Tenericutes; *Rhodopseudomonas palustris*, Alphaproteobacteria; *Bacillus cereus*, Firmicutes; *Actinomyces radicidentis*, Actinobacteria; *Bacteroides fragilis*, Bacteroides; *Treponema primitia*, Spirochaetes), Archaea (*Methanobrevibacter olleyae* and *Methanosphaera* sp. rholeuAM74, Euryarchaeota), Fungi (*Pleurotus ostreatus*, Basidiomycota; *Saccharomyces cerevisiae*, Ascomycota; *Blastocladiella emersonii*, Blastocladiomycota; *Spizellomyces punctatus*, Chytridiomycota; *Mucor circinelloides*, Mucoromycota), Plants (*Arabidopsis thaliana*, dicotyledons; *Oryza sativa* japonica, monocotyledons; *Selaginella moellendorffii*, Lycophytes; *Physcomitrella patens*, Bryophyta; *Pinus pinaster*, Gymnosperma) and animals (*Mnemiopsis leidyi*, Ctenophora; *Amphimedon queenslandica*, Porifera; *Pocillopora damicornis*, Cnidaria; *Saccoglossus kowalevskii*, Hemichordata; *Acanthaster planci*, Echinodermata; *Schistosoma mansoni*, Platyhelminthes; *Brachionus koreanus*, Rotifera; *Octopus bimaculoides*, Mollusca; *Platynereis dumerilii*, Annelida; *Caenorhabditis elegans*, Nematoda; *Macrobrachium nipponense*, Arthropoda/Crustacea; *Drosophila melanogaster*, Arthropoda/Hexapoda; *Branchiostoma belcheri*, Chordata/Cefalochordata; *Homo sapiens*, Chordata/Vertebrata/Mammals; *Gallus gallus*, Chordata/Vertebrata/Birds; *Alligator mississippiensis*, Chordata/Vertebrata/Reptiles; *Danio rerio*, Chordata/Vertebrata/Fishes and *Xenopus tropicalis*, Chordata/Vertebrata/Amphibians), which represent dataset 1 (Table S1). 

Additional animal GPx sequences were retrieved to further study the specific evolution of animal GPx For this analysis, we included sequences from the following animal species: *Dendronephthya gigantea*, *Nematostella vectensis*, *Pocillopora damicornis*, *Stylophora pistillata*, *Hydra vulgaris* (Cnidaria), *Helobdella robusta* (Annelids), *Pomacea canaliculata*, *Crassostrea gigas* (Molluscs), *Schistosoma japonicum* (Platyhelminthes), *Caenorhabditis briggsae* (Nematoda), *Lingula anatina* (Brachiopoda), *Limulus polyphemus*, *Camponotus floridanus*, *Papilio glaucus*, *Acyrthosiphon pisum*, *Macrobrachium nipponense* (Arthropoda), *Ciona savignyi* (Chordata/Tunicate), *Branchiostoma floridae* (Cephalochordata), *Acanthaster planci*, *Apostichopus japonicas*, *Strongylocentrotus purpuratus* (Echinoderms), *Oryzias latipes*, *Electrophorus electricus*, *Gadus morhua* (Fishes), *Nanorana parkeri* (Amphibians), *Picoides pubescens*, *Anas platyrhynchos*, *Columba livia* (Birds), *Chrysemys picta*, *Pantherophis guttatus*, *Thamnophis elegans*, *Crocodylus porosus*, *Gavialis gangeticus* (Reptiles), *Bos taurus*, *Sus scrofa*, *Mus musculus*, *Rattus norvegicus* and *Macaca mulatta* (Mammals)”, which represent dataset 2 (Table S1).

### 2.2. Sequence Alignments and Evolutionary Analyses

We performed sequence alignments of GPx coding sequences, using the MUSCLE algorithm [34], available within Molecular Evolutionary Genetics Analysis (MEGA) 7.0 software [35]. We trimmed the alignments manually in order to exclude the regions with ambiguous sites and extended gaps. The best fit evolutionary model used for both GPx phylogenetic trees was LG (Le Gascuel), with invariable sites and gamma-distributed rates, which was predicted on ProtTest 3 [36]. Phylogenetic trees were reconstructed by Bayesian inference, using BEAST 1.10.4 [37], or MrBayes 3.2.7a [38], employing BEAGLE library [39] in both cases, at CIPRES platform [40]. The birth and death model was selected as atree prior, and 70,000,000 generations (for BEAST), or 2,500,000 generations (for MrBayes) were performed with Markov chain Monte Carlo algorithm (MCMC) for evaluation of posterior distributions in all cases. The MCMC files convergence was verified with Tracer v.1.6 [41], and the consensus trees were generated using TreeAnnotator, available at BEAST package. The derived trees were edited and viewed using iTOL [42].

## 3. Results

### 3.1. Glutathione Peroxidases Have a Monomeric Common Ancestor with Peroxidatic Cysteine

**Figure 1:**
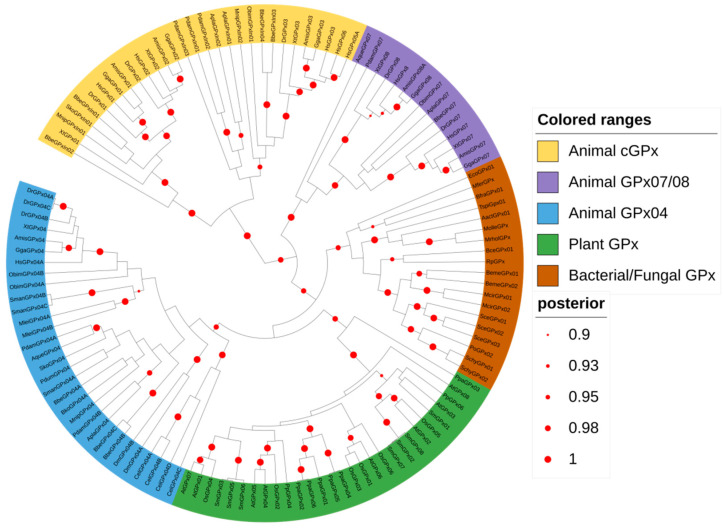
Global GPx tree of representative species from main taxonomic groups. Glutathione peroxidase protein sequences from major phyla of bacteria, fungi, plants, and animals were retrieved and used to reconstruct a phylogenetic tree, using Bayesian inference. A total of 124 sequences were used, and the tree was built on BEAST software, available at CIPRES platform. The posterior probabilities are discriminated according to the figure legend; only values above 0.9 are indicated.

Due to the differences in GPx across the major kingdoms of life, regarding their sequences and reducing agents, it is unclear whether the glutathione peroxidase genes derived from a common ancestor or genes from different origins converged to a similar function (functional convergence). To answer this question, we retrieved GPx protein sequences from major phyla of bacteria, fungi, plants, and animals, and we reconstructed a phylogenetic tree using the Bayesian method. This analysis supports the existence of a single GPx common ancestor, which eventually gave rise to all GPx subtypes, which in animals would give rise to monomeric GPx (GPx04 and GPx07/08 clades) and the classical GPx (cGPx) (Figure 1). Sequences from fungi, bacteria, and plants most likely derived from the same ancestor that gave rise to the animal GPx4 subfamily, which are mainly monomeric and devoid of the dimer/tetramic domain organization found in classical GPx (Figure 1). Moreover, non-bilaterial animals, such as sponges (e.g., Amphimedon queenslandica) or species from Ctenophora phyla (e.g., Mnemiopsis leidyi), considered as basal animals, only contain monomeric GPx (GPx04/07 and GPx04, respectively) (Table S1). Despite the described SeCys-GPx4 found in animals, the glutathione peroxidases from other kingdoms contain a peroxidatic cysteine (Cys_p_) in their catalytic site, which strongly suggests that the common ancestor of all GPx was a monomeric protein with a peroxidatic cysteine. This amino acid was eventually replaced by SeCys in animal lineage throughout the evolution but did not remain fixed in all phyla.

### 3.2. Selenocysteine Was Introduced into GPx Proteins Right from the Arise of Metazoa Kingdom

Most glutathione peroxidases from vertebrates contain a SeCys in their catalytic site, a feature that has not been seen in insects and nematodes. In order to determine the occurrence of this untypical amino acid within the GPx family, we searched and retrieved GPx sequences from several animal species, covering the major animal phyla (Table S1). The UGA codon that codes for SeCys (U), presented within the motif “NVAxxU”, is found since Porifera and Ctenophora phyla (non-bilateria) up to the vertebrates. This amino acid is present in most animal GPx04 and classical GPx protein sequences, with a few supposed reverse mutations to Cys (Table S1); however, all GPx sequences retrieved from nematode, Hexapoda, and mammal’s GPx5 subfamily do not contain SeCys in their active site, suggesting a selective pressure favoring Cys instead of SeCys in these groups. Besides, all GPx07/08 sequences throughout the animal kingdom lack selenocysteine. Collectively, these results suggest that the SeCys amino acid was incorporated into animal GPx sequences early in the evolutionary history of metazoans, and it was kept in most GPx04 and cGPx protein sequences throughout their evolution. 

### 3.3. Animal GPx Proteins Are Evolutionarily Classified into Three Main Groups 

**Figure 2:**
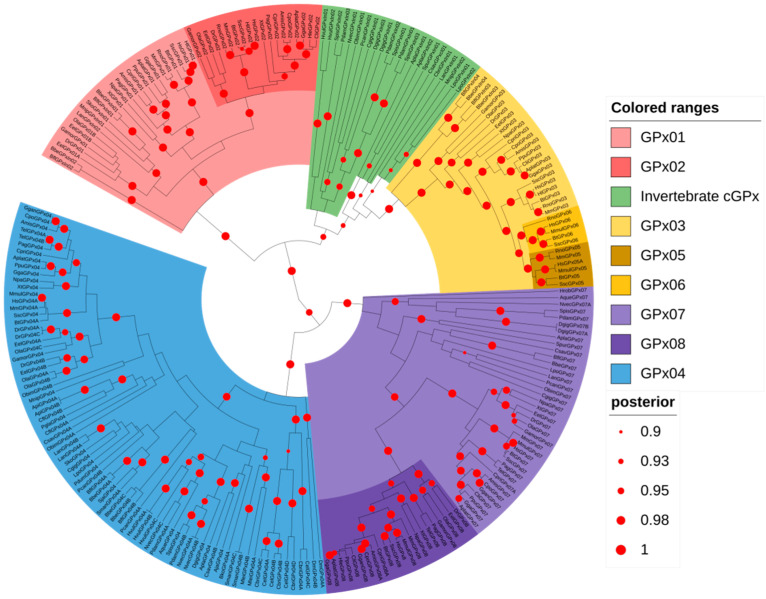
Focused GPx tree of representative animal sequences. Glutathione peroxidase protein sequences from animals were retrieved and used to reconstruct a phylogenetic tree, using Bayesian inference. A total of 241 sequences were used, and the tree was built on BEAST software, available at CIPRES platform. Green branches display the invertebrate classical GPx sequences. The posterior probabilities are discriminated according to the figure legend; only values above 0.9 are indicated.

To explore the phylogenetic relationships among the animal GPx, we reconstructed a second phylogenetic tree, expanding the count of animal sequences and species (Figure 2). This analysis reveals two main well-supported groups: one containing monomeric GPx sequences (monomeric GPx group) and another one with the classical GPxs (classical GPx group). The monomeric GPx group includes two subgroups, which correspond to GPx04 (blue) and GPx07/08 (purple) proteins. Orthologs of vertebrate GPx04 and GPx07/08 are found in many invertebrates; however, the rise of the GPx08 gene seems to come from a gene duplication from GPx07 in the vertebrate’s common ancestor (Figure 2). Moreover, none of the invertebrate GPx07/08 orthologous sequences contain the characteristic short cytosolic loop, followed by a single transmembrane domain, found in the vertebrate GPx08 N-terminal protein sequences [18], reinforcing that they are closely related to GPx07 and that the GPx08 subfamily is vertebrate-specific (data not shown).

The classical GPx group is also divided into smaller clades: one containing sequences belonging to GPx01/02 subfamilies, and the other one sequences from the GPx03/05/06 subfamilies (Figure 2). In agreement with other studies [7,43], GPx05 and GPx06 subfamilies came from a common ancestor of GPx03 gene duplication in mammals (dark yellow). GPx02 (dark red) also comes from another gene duplication but from GPx01 in a vertebrate’s common ancestor (Figure 2). Our analysis regarding the classical GPx sequences from invertebrates showed conflicting results between the trees constructed by BEAST and MrBayes software. On BEAST, most of these sequences were grouped into the GPx03/05/06 clade (Figure 2); however, the animal GPx tree generated using MrBayes grouped these sequences into the GPx01/02 clade (Figure S1). This inconsistency is probably due to the amino acid composition in these sequences, which has some similarity to both cGPx clades (data not shown). Therefore, we classified them as classical GPx from Invertebrates (cGPxIn), as they contain the dimeric/tetramic domain composition and were grouped into the wider classical GPx group (Figure 2). Despite this disagreement, a few sequences from Brachiopoda, Crustacea, and Hemichordata (LanGPxIn01, MnipGPxIn01, and SkoGPxIn01, respectively) grouped into the GPx01/02 clade in both phylogenetic trees. Moreover, sequences from *Branchiostoma belcheri* and *Branchiostoma floridae* (Cephalochordata) grouped into both GPx01/02 (BbeGPxIn01, BbeGPxIn02, BflGPxIn01, BflGPxIn02), and GPx03/05/06 (BbeGPxIn03, BbeGPxIn04, BflGPxIn03, BflGPxIn04) clades (Figure 2). Hence, the presence of both subdivisions of cGPx is already seen in Cephalochordata that are considered as basal lineage with respect to all vertebrates.

### 3.4. The Radial Spreading of GPx in Animals

**Figure 3:**
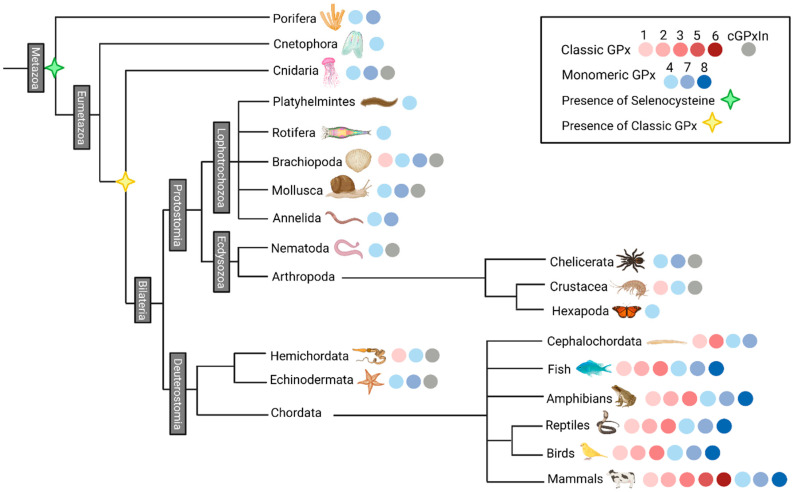
Presence of each type and subtype of glutathione peroxidase proteins found in major animal phylum, or subphylum. Red colors: classical GPx; blue colors: monomeric GPx; gray color: classical GPx from invertebrates (cGPxIn); green star: introduction of selenocysteine at the GPx catalytic site; yellow star: supposed arising of classical GPx. Figure created with BioRender.com.

Based on the animal GPx sequences collected and on the animal-focused GPx phylogenetic tree (Table S1 and Figure 2), we analyzed the prevalence of each GPx subfamily found in the main animal phyla (Figure 3). The canonical GPx04 has at least one sequence present in most species, and all animal phyla. It is the most conserved GPx and it is the only subfamily found in Cnetophora, Rotifera, Platyhelmintes, and in the subphylum Hexapoda (Figure 3). The monomeric Cys-exclusive GPx07 is found in porifera and many eumetazoas species. Interestingly, this subfamily of GPx is not present in most worms or worm-like phyla, such as Nematoda, Platyhelmintes, Rotifera, and Hemichordata (Figure 3). Finally, the presence of classical GPx is found since cnidaria, and it was lost in a few phyla; however, it is found in the genomes of all deuterostomic species (Figure 3). Furthermore, in Chordata, these genes evolved into more classical GPx subdivisions, eventually resulting in five different cGPx subfamilies, found in mammals. Together, these results indicate that both GPx07 and cGPx are present in the genome of invertebrates before the bilateria, and they were lost in a few phyla.

## 4. Discussion

There is large structural diversity observed among the glutathione peroxidase enzymes found across all kingdoms of life. Perhaps, the only main characteristic that brings them all together is the ability to reduce peroxides into water and alcohols. Throughout a long-term evolution, their structure, as well as their catalytic mechanisms and substrate preferences diversified at a point that GPxs are no longer considered to be simple ROS scavengers. There is strong evidence that GPxs can act as ROS sensors and are able to modify other proteins regulating their function [44,45,46], as well as being linked to many biological processes that lead to different cell fates [8]. Therefore, the evolutionary study of these genes might bring new perspectives on how this family has evolved and how antioxidant enzymes diversified within lineages.

Previous studies have claimed that GPx genes came from a common ancestor that was a member of PHGPxs but failed to obtain significant statistical support [7,47]. Applying Bayesian inference, we reconstructed a global phylogenetic tree using sequences from the major bacterial, fungal, plant, and animal phyla that provided a high posterior score, suggesting that all GPxs from these kingdoms came from a common ancestor gene. In addition, all non-animal GPxs share the same common ancestor with the animal GPx04, a SeCys-GPx. This result strengthens the hypothesis that SeCys was specifically incorporated into animal GPx, and not lost in other kingdoms as previously suggested [17]. Likewise, along with the animal GPx tree, it proposes that the animal Cys-exclusive GPx07 came from gene duplication from the monomeric animal GPx ancestor, and likely it underwent a reverse mutation to Cys.

The presence of genes encoding classical GPx and GPx07 in invertebrates was previously described in a few phyla [12,21,33], but it was unclear how these genes were distributed across the animal kingdom. Through sequence search and analysis, we detected the presence of these GPx subtypes in several invertebrate phyla, suggesting that GPx07-like genes were established in the early history of metazoan, while cGPx must have preceded bilateria divergence. In addition, we believe these particular GPx subtypes might have been independently lost in some species, while SeCys-to-Cys reverse mutations in GPx04 also occurred in some invertebrates. Our data consolidate the concept that classical GPxs and SeCys-GPxs are not exclusive to vertebrates, contradicting a hypothesis previously accepted due to the scarcity of invertebrate’s genomes sequenced [7].

**Figure 4:**
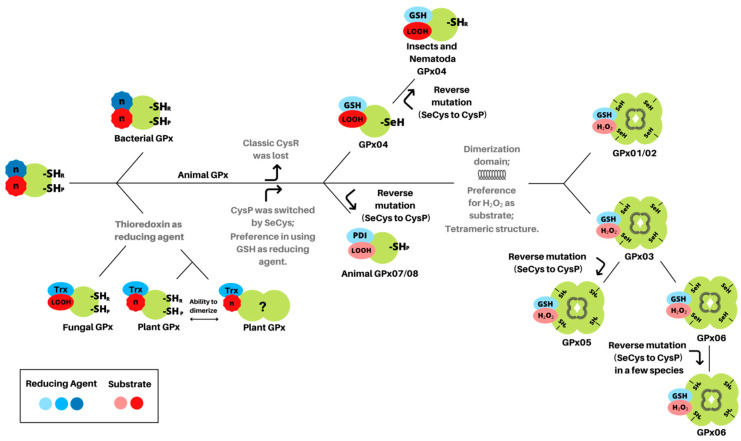
Diversification of glutathione peroxidases. The ancestral GPx protein was a promiscuous enzyme, regarding its substrate and reducing agent, and it contained a peroxidatic (SH_P_) and resolving cysteines (SH_R_). Throughout evolution, it has independently evolved to use specific substrates and reducing agents. Further information is described within the “Discussion” section. Reducing agents and substrates displayed indicate preferences, not necessarily exclusivity. GSH: reduced glutathione; Trx: thioredoxin; PDI: protein disulfide isomerase; n: several substrates/reducing agents; LOOH: lipid peroxides; H_2_O_2_: hydrogen peroxide; SeH and SeCys: selenocysteine; CysR: resolving cysteine; CysP: peroxidatic cysteine.

We propose now a model on how GPx proteins have diversified and evolved (Figure 4). Initially, the enzyme encoded by the ancestral gene would be monomeric, containing both peroxidatic and resolving cysteines, and promiscuous regarding its reducing agents and substrates, similar to what is seen in bacteria [29]. In fungi and plants, the GPx gene must have duplicated a few times, eventually culminating in enzymes with distinguished subcellular localization and preference for thioredoxin over GSH as a reducing agent [24]. In fungi, although also acting as a redox sensor, all GPxs seem to play a role in the reduction in lipid peroxides [23,25]. This activity over membrane peroxides can be considered the most ancient activity of GPx gene in its history, as it can also be observed in the correspondent GPx in animals (GPx04) and in plant GPxs as well [27]. Furthermore, lipid peroxidation is a chain oxidation process, which can lead to severe cell damages [48], and therefore, it might cause a stronger selective pressure on this specific GPx activity [25].

Plant GPxs reduce a wide range of substrates, such as cumene hydroperoxide, H_2_O_2_, and lipid peroxides, and they collectively prefer thioredoxin as a reducing agent [27,49]. Intriguingly, some plant GPx are able to form homodimers, even without the dimer/tetrameric interfaces found in classical GPxs from animals [50]. It has been shown that dimerization is stabilized by several aromatic residues, or via intermolecular disulfide bonds [51]; however, it is not required for the peroxidase function or thioredoxin interaction; nevertheless, it was suggested to be important to reduce large/complex peroxides, such as the aromatic ones [52]. 

The GPx proteins underwent great diversification and we, therefore, propose a model as an attempt to summarize the main milestones of this process (Figure 4). Firstly, the peroxidatic cysteine was exchanged by a SeCys. This substitution probably has happened early in metazoan history, since GPx sequences from choanoflagellata protists, which strongly resemble choanocytes (sponge cells), lack this amino acid residue (Table S1). This modification not only increased the GPx reactivity towards H_2_O_2_ but also removed the need for resolving cysteine, which eventually was also lost. The animal GPx common ancestor would give rise to three different types of GPx. The closest to non-animal GPx, GPx04, retained its monomeric structure and catalytic capacity to reduce lipid peroxides [16,53]. Despite the absence of the amino acid residues responsible to bind to GSH, the presence of selenocysteine in the GPx04 contributed to the use of glutathione as a reducing agent [8,19]. In addition, in nematodes and insects, the GPx04 SeCys residue was exchanged back to Cys, apparently by a reverse mutation, an evolutionary event that also has happened to mammals GPx05 and some GPx06 [43].

The animal GPx07/08 were established as enzymes targeted to the endoplasmic reticulum. These GPxs subfamilies contain a peroxidatic Cys residue instead of the SeCys in their catalytic site, and they lack a classical resolving Cys. Briefly, the SeCys loss decreased the reactivity of GPx07/08 towards GSH, allowing competitive oxidation of PDI and GSH in the ER, finely regulating the redox buffer and contributing to the oxidative protein folding [8,18]. Likewise, GPx07/08 was proposed to act by protecting cell membranes from peroxides [19,21], however, further studies must be performed to confirm this activity. 

Ultimately, the most recent classes of glutathione peroxidases comprise those that were first described as GPxs, and contain the dimeric/tetrameric domains [6]. The homotetrameric structure, combined with four arginine residues and one lysine residue, are thought to be responsible for the specificity/preference towards GSH and H_2_O_2_ [54], although there are variations in these amino acids in different groups of cGPxs [8]. The SeCys-to-Cys reverse mutation in classical glutathione peroxidases seems to be a rare event, probably due to the protection of the highly reactive SeCys residues within the quaternary structure. In our data, besides GPx05 and some GPx06 from mammals, only GPx02 members of Cephalochordata and cGPxIn from Nematoda present the Cys residue (Table S1), reinforcing that these events occur independently throughout the evolution of GPxs [43]. The classical GPxs are also under a stronger positive selection in mammals than the monomeric ones, probably due to the functional constraints found in GPx04/07/08 that defend the cell against oxidative damage in membranes. The residues under positive selection found in mammalian cGPxs are close to the active or dimeric sites and can eventually modify the catalytic activity or substrate biding [43]. Further studies must be conducted to fully understand why these classes are expanding and diversifying in Chordata, but we believe it could be related to the fine-tuning of the countless biological processes that take place within these species, which can be further modulated by the ROS metabolism [8,18].

Many human diseases cause a redox imbalance and decrease GPx activity, which culminates in the further development of the condition [55,56]. By understanding how these enzymes function and which substrates they prefer, it is possible to come up with rational strategies to enhance their activity. The supplementation of selenium to increase GPx production and activity [57], for example, would potentially only affect half of the human glutathione peroxidases, since the other half does not contain this amino acid. This information might interfere with the decision about the supplement to select clinically, or in which molecules or substances researchers must focus their efforts. Furthermore, although non-animal GPxs use other reducing agents, all human GPxs have evolved to use reduced glutathione as substrate. This means that the supplementation with GSH would not only rebalance the antioxidant content per se [58], but also potentially boost the activity of all GPxs by providing one of its substrates.

## 5. Conclusions

Glutathione peroxidase genes came from a common ancestor that diversified independently in different kingdoms. In early animal evolution, selenocysteine was introduced into GPx catalytical site, and three GPx classes emerged: (a) the most similar to non-animal GPxs and present in most species, GPx04; (b) the monomeric Cys-exclusive, found since porifera, GPx07; (c) the homotetrameric GPx, which is found since cnidaria and heavily expanded its subfamilies in vertebrata, cGPx. Nevertheless, the loss of some classes of GPx, as well as the SeCys-to-Cys reverse mutation occurred independently in different animal phyla. The evolution of glutathione peroxidases seems to be an ongoing process, which leads to the ongoing diversification of their functions. 

## Data Availability

Not applicable.

## Supplementary Materials

The following are available online at https://www.mdpi.com/article/10.3390/biology10111165/s1, Figure S1: GPx tree of representative animal sequences constructed on MrBayes; Table S1: Datasets.
