# Supplementary material for: Going Forward and Back: The Complex Evolutionary History of the GPx
Source: Biology (Basel). 2021 Nov 12;10(11):1165. doi: 10.3390/biology10111165 (PMC8614756; doi:10.3390/biology10111165)
Supplement: Supplementary file 1 [file biology-10-01165-s001.zip › Figure S1.pdf]

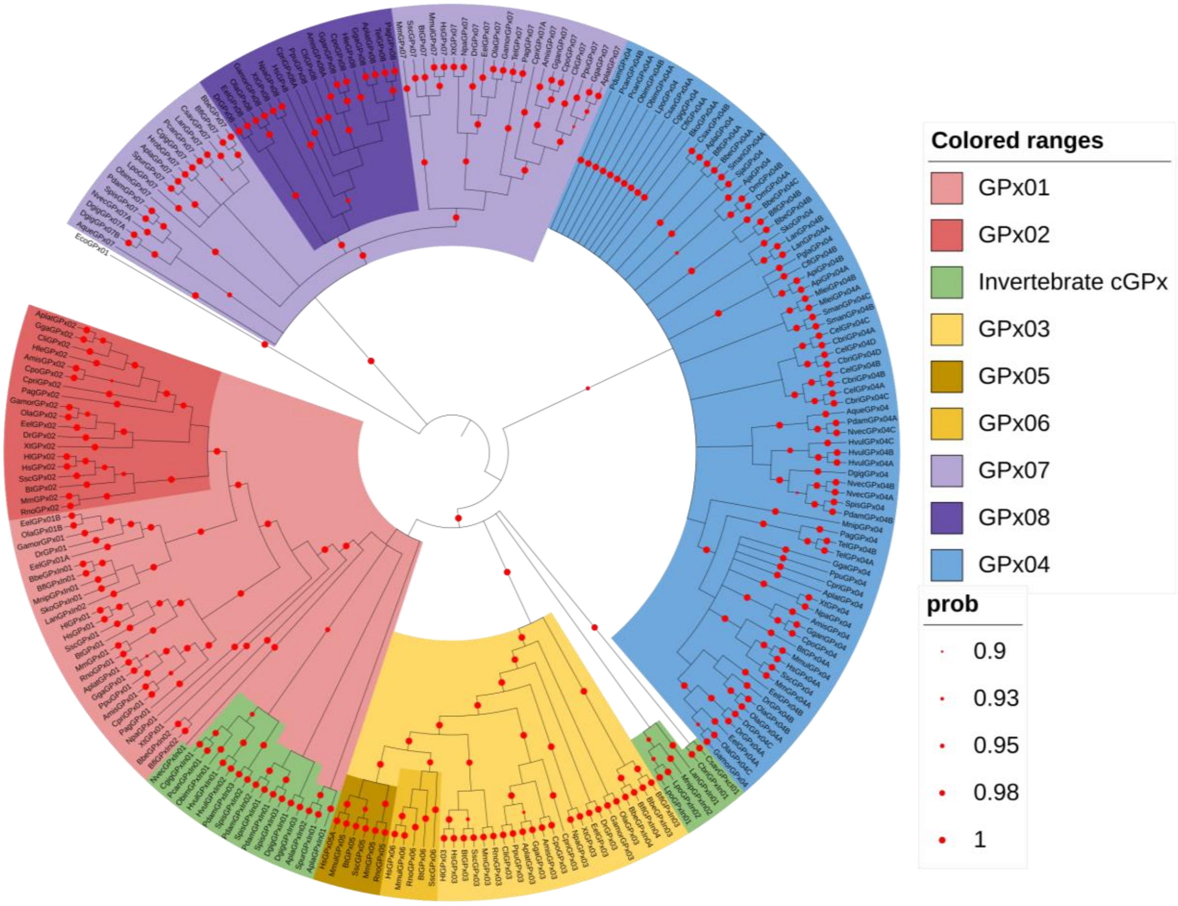

Figure S1. Focused GPx tree of representative animal sequences constructed on MrBayes. Glutathione peroxidase protein sequences from animals were retrieved and used to reconstruct a phylogenetic tree, using Bayesian inference. A total of 241 animal sequences, plus the *E. coli* GPx sequence, were used and the tree was built on MrBayes software, available at CIPRES platform. Green branches display the invertebrate classical GPx sequences. The posterior probabilities are discriminated according to the figure legend; only values above 0.85 are indicated.
